# Enrichment of circulating trophoblasts from maternal blood using laminar microscale vortices

**DOI:** 10.1002/pd.5901

**Published:** 2021-02-01

**Authors:** Ann‐Sophie Vander Plaetsen, Jana Weymaere, Olivier Tytgat, Magaly Buyle, Dieter Deforce, Filip Van Nieuwerburgh

**Affiliations:** ^1^ Laboratory of Pharmaceutical Biotechnology Ghent University Gent Belgium; ^2^ Department of Life Science Technologies IMEC Leuven Belgium; ^3^ Department of Obstetrics and Gynecology Ghent University Hospital Gent Belgium

**Keywords:** Cell‐free DNA < fetal cells, nucle, ic acids & proteins, prenatal diagnosis, fetal cells < fetal cells, nucleic acids & proteins, noninvasive prenatal testing < fetal cells, nucleic acids & proteins, size‐based enrichment, Y‐chromosome qPCR, Y‐chromosome STR analysis

## Abstract

**Objective:**

Enrichment of circulating trophoblasts (CTs) from maternal blood at week 11–13 of gestation, using laminar microscale vortices, and evaluation of the performance of the VTX‐1 Liquid Biopsy System in terms of CT recovery and purity.

**Method:**

Eight mililiter of blood was collected from 15 pregnant women and processed with the VTX‐1 Liquid Biopsy System. Y‐chromosome specific quantitative PCR was performed to estimate the number of enriched male CTs. To evaluate the VTX‐1 performance, the target cell recovery was characterized by spiking experiments with a trophoblast cell line. Furthermore, the total quantity of DNA after enrichment was used to calculate the number of retained maternal cells.

**Results:**

Successful recovery of male CTs was established in 7 out of 10 first trimester samples from pregnant women carrying a male fetus. The number of CTs, recovered from 8 ml of blood, was estimated between two and six. Spiking experiments resulted in a CT recovery of ±35 % with ±1524 retained maternal blood cells.

**Conclusion:**

CTs can be enriched from maternal blood with high purity, using laminar microscale vortices, starting from 8 ml of blood.

## Key Points

What's already known about this topic?

- Cell‐free noninvasive prenatal testing (cfNIPT) is an established, clinically validated method for the prenatal screening of large chromosomal aberrations.
- The isolation of circulating trophoblasts (CTs), allowing cell‐based NIPT, has been accomplished by means of marker‐based enrichment, although approximately 30‐40 ml of maternal blood is required.
- Size‐based enrichment of CTs was published in 2012, but was never repeated nor confirmed.

What does this study add?

- Laminar microscale vortices allow size‐based enrichment of circulating trophoblasts, starting from only 8 ml of maternal blood.
- CT recovery and purity after enrichment using the VTX‐1 Liquid Biopsy System are reported.

## 1 INTRODUCTION

Efficient prenatal genomic‐based testing is essential for pregnant women to make informed reproductive decisions.^1^ Since 2011, cell‐free noninvasive prenatal testing (cfNIPT) has been clinically implemented as a first trimester screening test for Trisomy 21, 18, and 13.^2^, ^3^, ^4^, ^5^, ^6^, ^7^ The increased use of cfNIPT has led to a large reduction of invasive diagnostic procedures such as amniocentesis and chorionic villus sampling.^8^ cfNIPT is based on massively parallel sequencing of a small fraction of cell‐free fetal DNA fragments in the presence of a large fraction of cell‐free maternal DNA.^7^, ^9^, ^10^ Therefore, large genetic aberrations, such as aneuploidies, can be detected but smaller genetic aberrations, such as single‐gene disorders caused by single nucleotide polymorphisms (SNPs) are far more challenging.^11^, ^12^ As an alternative, several research groups have engaged in the quest for the isolation of fetal cells from the blood stream of pregnant women to allow cell‐based non‐invasive prenatal testing (cbNIPT) during the first trimester of pregnancy.^13^, ^14^, ^15^ As cbNIPT allows genetic analyses of the fetal genome in absence of contaminating maternal DNA, cbNIPT could be a valuable alternative for the current cfNIPT assay.

Different circulating fetal cell types such as nucleated red blood cells (nRBCs), trophoblasts, and lymphocytes have been reported in the past.^16^, ^17^ Unfortunately, all types of fetal cells are fragile and extremely rare with a frequency of approximately one fetal cell in 10^9^ maternal blood cells.^16^ Current cbNIPT methods are mainly focused on the isolation of circulating trophoblasts (CTs) because of the presence of relatively specific trophoblast markers^18^ and their potentially larger cell size (>15 µm) compared to other maternal blood cells.^15^, ^19^ Numerous approaches have been investigated regarding the isolation of CTs from maternal blood, but only a few research groups have made significant progress in the quest for a feasible cbNIPT workflow.^13^, ^14^, ^15^

The Danish research group Arcedi Biotech (Vejle, Denmark) discovered a unique combination of trophoblast markers through the characterization of CTs by means of microarray analysis.^18^ Their results indicate that CTs express both the ectodermal marker Cytokeratin and the mesenchymal marker CD105. Based on these findings, in 2016, Arcedi Biotech succeeded in the enrichment of CTs with positive magnetic‐activated‐cell‐sorting (MACS), using the surface marker CD105.^20^ After enrichment, Cytokeratin positive CTs were selected and isolated by means of immuno‐fluorescent staining. Finally, a recovery of on average 0.42 CTs/ml starting from 30 ml of maternal blood was reported.^20^ Marker‐based enrichment of CTs from maternal blood was also reported recently by Vossaert et al.^21^ from Baylor college (Houston, USA). This research group demonstrated the identification of on average 0.2 putative CTs/mL in 30 ml of maternal blood using positive MACS enrichment on 95 validation cases.^21^ Antibodies against HLA‐G, TROP‐2, and EpCAM were used instead of anti‐CD105. Both aforementioned research groups demonstrated the successful isolation of trophoblasts from maternal blood. However, the described methods are still time consuming and complex, and they include several pre‐enrichment procedures, such as red blood cell lysis and formaldehyde fixation. Moreover, a high volume of maternal blood is required to allow the recovery of CTs in all maternal samples.

Besides marker‐based enrichment, size‐based enrichment can also be performed by exploiting the larger cell size of some of the CTs compared to other maternal blood cells. In 2012, the group of Paterlini‐Bréchot from Rarecells Diagnostics (Paris, France) published promising research demonstrating the correct diagnosis of 63 fetuses at risk for cystic fibrosis or spinal muscular atrophy using size‐based CT enrichment.^15^ In this workflow, trophoblast cells were enriched from maternal blood by means of their in‐house developed Isolation by Size of Epithelial Trophoblastic cells (ISET) technology (Rarecells Diagnostics). With this ISET technology, blood is filtered over 8 µm pores. Next, putative trophoblasts larger than 15 µm are isolated individually. An average of 1–2 CTs/ml in a total 10 ml of maternal blood was reported using this ISET technology.^15^ Unfortunately, these promising results have not been replicated nor have additional papers been published reporting successful size‐based isolation of trophoblasts.

**FIGURE 1:**
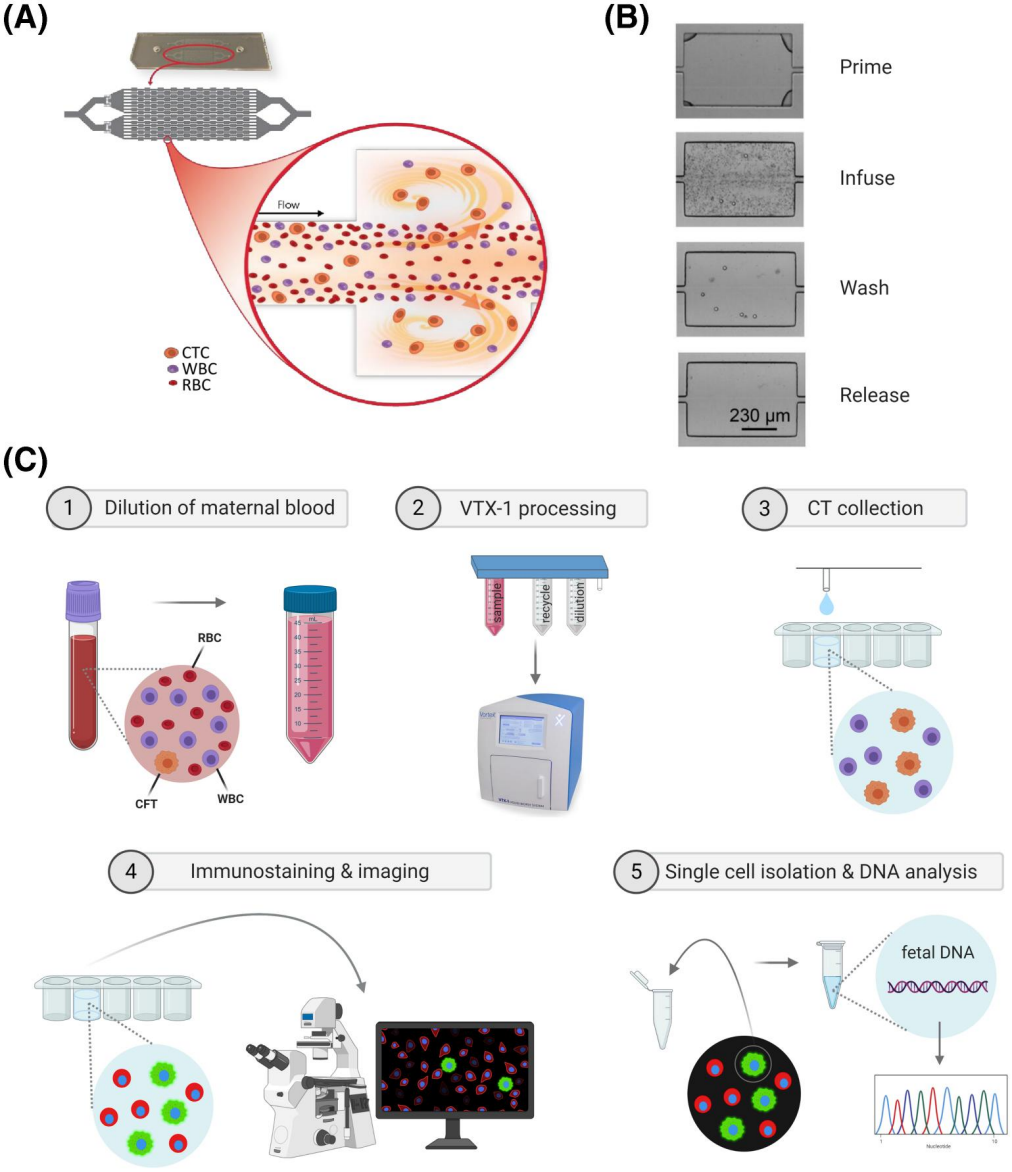
Vortex technology and the integration in a cell‐based non‐invasive prenatal testing (cbNIPT) workflow. (A) Each vortex microfluidic chip contains two devices. Each device consists of 16 parallel channels with nine serial reservoirs. Large target cells, such as circulating tumor cells (CTCs) and CTs, are trapped in the reservoirs via micro‐scale vortices, while unwanted red blood cells (RBCs) and white blood cells (WBCs) flow off the chip. (B) VTX‐1 enrichment. Microfluidic channels are first primed with PBS to remove air bubbles and vortices are developed in all reservoirs. Blood is then injected and large target cells are captured in the reservoirs. The device is washed with PBS and trapped target cells are finally released from the reservoirs (A and B are adapted from^25^, ^30^ with Vortex Biosciences' authorization). (C) Integration of the Vortex technology in a beginning‐to‐end cbNIPT workflow. (Created in BioRender.com)

Recently, many novel size‐based enrichment technologies have been developed for the isolation of Circulating Tumor Cells (CTCs) in the field of cancer liquid biopsy. These new technologies can potentially be used for the isolation of CTs from maternal blood, since both CTCs and CTs demonstrate a larger cell size. Moreover, as trophoblasts are the largest and most abundant type of circulating fetal cells, it is most likely that these are enriched from maternal blood using size‐based methods.^16^ A promising innovative technology is the automated VTX‐1 Liquid Biopsy System (Vortex Biosciences, Pleasanton, USA). In contrast to microfilter technologies such as ScreenCell,^22^ ISET,^23^ or Parsortix,^24^ this Vortex technology is based on inertial microfluidics and laminar microscale vortices^25^, ^26^, ^27^ for the enrichment of larger cells. After infusion of the diluted blood sample on the proprietary Vortex high‐throughput chip, large cells, including CTs, are trapped in reservoirs via micro‐scale vortices, based on size, shape, and deformability. Smaller cells, such as unwanted RBCs and most white blood cells (WBCs) continue in the main stream, flowing off the chip (Figure 1A, B). Finally, after washing away any residual blood cells, the trapped target cells are released from the microscale vortices in the reservoirs and are collected in a recipient of choice. No loss of target cells is expected during these on‐chip washing steps.

In contrast to the established cbNIPT methods, VTX‐1 enrichment allows the processing of whole blood without pre‐enrichments procedures such as formaldehyde fixation, red blood cell lysis, or density gradient centrifugation. Therefore, no sample manipulations are required before enrichment and consequent target cell loss is prevented. Figure 1C illustrates how the Vortex technology could be integrated in a beginning‐to‐end cbNIPT workflow. After VTX‐1 enrichment, immunostaining and imaging can be performed to select putative CTs. Finally, single CTs can be isolated, the fetal genome can be amplified, and genetic analysis can be performed.

In this study, the enrichment of CTs from 8 ml of maternal blood by means of laminar microscale vortices is assessed at 11–13 weeks of gestation. The retrieval of male trophoblasts is determined using Y‐chromosome specific quantitative PCR (qPCR). Furthermore, to evaluate the performance of the VTX‐1 Liquid Biopsy System, both the recovery of VTX‐1 enrichment and the purity of the enriched CTs are estimated. To calculate the recovery, spiking experiments with a trophoblast cell line were performed.

## 2 METHODS

### 2.1 Sample collection

Maternal blood samples were collected at Ghent University Hospital at 11–13 weeks of gestation. Each blood sample contained 12 ml maternal blood, collected in K_3_EDTA blood collection tubes (Thermo Fisher Scientific). All pregnant women were adult and signed informed consent. Ethical approval was obtained from the ethical review board of Ghent University Hospital, B670201524235. All methods were performed in accordance with the Declaration of Helsinki.

### 2.2 Fetal sex determination from plasma

To determine the fetal sex based on circulating cell‐free DNA in maternal plasma, 4 ml of maternal blood was processed within 4 h after blood collection. Following density gradient centrifugation for 15 min at 1200 x g, 400 µl of plasma was collected and cell‐free DNA was extracted with the QIAamp DNA Blood Mini Kit (Qiagen, Hilden) following manufacturer's recommendations. DNA was eluted in 100 µl nuclease‐free water at 65°C. Next, as described by Picchiassi et al.^28^ quantitative PCR (qPCR) was executed to detect the Y‐chromosome specific multicopy *DYS14* sequence located within the *TSPY* gene. Amplification was conducted in a 50 µL volume, containing 30 µL extracted DNA, 200 µM of each dNTP (Thermo Fisher Scientific), 1 X PCR buffer (Qiagen), 1.25 µM EvaGreen dye in aqueous solution (VWR), 2.5 U HotStarTaq DNA polymerase (Qiagen), and 0.9 µM of both forward (5′ GGGCCAATGTTGTATCCTTCTC) and reverse (5′ GCCCATCGGTCACTTACACTTC) primer (Integrated DNA Technologies). The thermal cycling program starts at 95°C for 15 min, followed by 50 cycles at 95°C for 30 s, 57°C for 45 s, and 72°C for 1 min. 1 ng 2800M male control DNA (Promega, Madison, USA), 30 µL plasma from a woman carrying a female fetus, and 30 µl sterile nuclease‐free water were included in each run. C_q_ values were determined with LinRegPCR version 2020.0.^29^ A woman was considered to be carrying a male fetus when plasma C_q_ values were below 35.

### 2.3 Vortex enrichment

Maternal blood samples from ten women carrying a male fetus and five women carrying a female fetus were used for Vortex enrichment. From each woman, at least 8 ml of blood was processed on the fully automated VTX‐1 Liquid Biopsy System (Vortex Biosciences) within 12 h after blood collection. First, 8 ml of maternal blood was diluted ten times with PBS (Thermo Fisher Scientific). Next, the diluted blood sample was injected into the microfluidic chip at a flow rate of 8 ml/min and target cells were trapped in the vortices. After 2.5 h, a final volume of 1500 µl, containing the enriched target cells, was collected in an Eppendorf tube.

### 2.4 Detection of male cells after vortex enrichment

After the enrichment of target cells, DNA was extracted with the QIAamp DNA Blood Mini Kit. The extracted DNA was eluted in 50 µl sterile nuclease‐free water at 65°C, followed by evaporation to a final volume of 30 µl. Next, qPCR was performed on this extracted DNA to detect the Y‐chromosome specific multicopy *DYS14* sequence located within the *TSPY* gene, as previously described. A positive control, containing 1 ng 2800M Control DNA (Promega), and a negative control, consisting of 30 µl sterile nuclease‐free water, were included in each run.

To confirm the qPCR results, Y‐chromosome short tandem repeat (STR) analysis was performed in parallel on four maternal blood samples. For these samples, 16 ml of blood was processed instead of 8 ml using the VTX‐1 device. Half of the enriched cells were used for Y‐chromosome‐specific qPCR, while the other half was used for Y‐chromosome STR analysis. DNA was extracted as previously described, followed by evaporation to a final volume of 30 µl. Y‐chromosome STRs were amplified with the Investigator Argus Y‐12 QS Kit (Qiagen), following manufacturers recommendations. A positive control, consisting of 1 ng 2800M Control DNA (Promega), and a negative control, consisting of 10 µl sterile nuclease‐free water, were included in each run. After capillary electrophoresis of the STR‐amplicons with the ABI3130xl Genetic Analyzer (ThermoFisher Scientific), STR profiles were acquired and further analyzed with the GeneMapper ID‐x 1.2 software (ThermoFisher Scientific).

### 2.5 qPCR standard curve

To estimate the number of recovered male CTs after VTX‐1 enrichment, a dilution series of male reference DNA was analyzed, using defined concentrations of 2800M male control DNA (Promega), ranging from 20.00 pg/µl to 0.16 pg/µl. Furthermore, to mimic the VTX‐1 enriched samples, 8 ng female DNA was added to all calibrator dilutions. This female DNA was obtained from blood from women carrying a female fetus, using the QIAamp DNA Blood Mini Kit, following manufacturer's recommendations. Negative control samples containing 8 ng female DNA were also included to detect non‐specific amplification. These samples had C_q_ values of 41.99 ± 6.79 (*n* = 3). Sterile nuclease‐free water was used as no template control (NTC) to detect contamination. The NTC samples had C_q_ values above 50 (*n* = 3). The standard curve was generated in triplicate. The C_q_ values of all standard calibrator samples, the 8 ng female DNA negative control samples, and the NTC samples are shown in Supplementary Table 1. The standard curve, *R* ^2^, intercept, and slope are shown in Supplementary Figure 1.

### 2.6 Cell culture

Cells from a human choriocarcinoma JEG‐3 HTB‐36 cell line were obtained from the American Type Culture Collection (ATCC) and used for recovery calculation of VTX‐1 enrichment. These adherent epithelial cells, derived from the placenta, were chosen for their comparable characteristics as authentic circulating trophoblasts. The JEG‐3 cells were maintained in Eagle's Minimum Essential Medium (ATCC) supplemented with 10 % fetal bovine serum (Gibco, Thermo Fisher Scientific) and a mix of penicillin and streptomycin (Gibco, Thermo Fisher Scientific) at a final concentration of 100 units/mL and 100 µg/ml, respectively. The cells were cultured in an humidified atmosphere containing 5 % CO_2_ at 37°C.

### 2.7 Performance of VTX‐1 Liquid Biopsy System

All JEG‐3 cells used for recovery calculation, were pre‐stained with Hoechst 33342 Ready Flow^TM^ Reagent (Invitrogen) for 25 min. Target cell loss caused by VTX‐1 enrichment was estimated by spiking an exact number of pre‐stained JEG‐3 cells, ranging from 21 to 3389, in 4 ml of maternal blood before further dilution with 36 ml of PBS. After VTX‐1 enrichment, all retrieved Hoechst‐positive JEG‐3 cells were counted in flat bottom wells using a Zeiss Fluorescence Microscope (Carl Zeiss) and VTX‐1 recovery was calculated.

The number of retained maternal blood cells was estimated by determining the total quantity of DNA after VTX‐1 enrichment of 8 ml of maternal blood. After DNA extraction, the total DNA yield of three random samples was quantified with Qubit dsDNA High Sensitivity Assay kit (Life Technologies), following manufacturers recommendations. An estimate of the number of enriched, contaminating maternal cells was then made by dividing this DNA yield by 6 pg per human diploid cell.

## 3 RESULTS

### 3.1 Enrichment of trophoblasts from maternal blood using VTX‐1 enrichment

After Y‐chromosome‐specific qPCR‐based fetal sex determination on maternal plasma, 15 blood samples from pregnant women were processed on the VTX‐1 Liquid Biopsy System. Ten women were carrying a male fetus (samples 1–10) and five women were carrying a female fetus (samples 11–15). The plasma C_q_ values are listed in Supplementary Table 2. The fetal sex in all samples was confirmed by Ghent University Hospital, based on the cfNIPT results.

**FIGURE 2:**
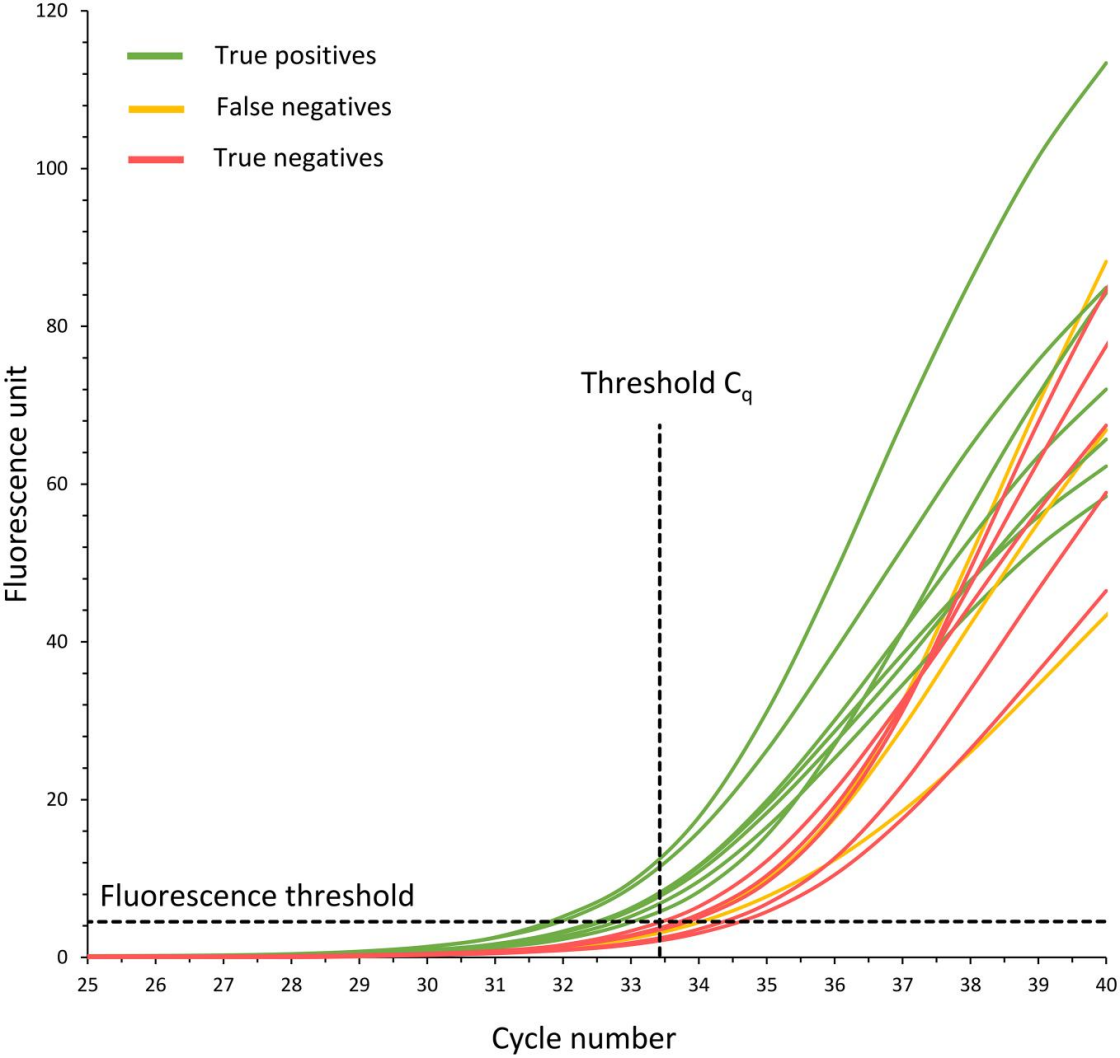
Fluorescent signal plotted against the quantitative PCR (qPCR) cycle number. Samples from women carrying a male fetus with a C_q_ value below or above the threshold C_q_ value are indicated in green (true positives) or orange (false negatives), respectively. Samples from women carrying a female fetus are indicated in red (true negatives). The fluorescence threshold and the threshold C_q_ are indicated by black dotted lines

After VTX‐1 enrichment of these 15 samples, Y‐chromosome‐specific qPCR was performed to assess the recovery of male trophoblasts in samples 1–10, while samples 11–15 were used to determine a threshold C_q_. For this assay, the threshold C_q_ value is defined as the lower limit of the 95 % confidence interval (33.39; 34.28), of the five true negative samples 11–15. A threshold C_q_ is required to distinguish the signal caused by the presence of male cells from the signal caused by non‐specific amplification. Seven out of 10 male fetus samples show C_q_ values below 33.39, indicating the presence of male cells in the pool of enriched cells. These samples are therefore considered as true positives. Three out of 10 male fetus samples (samples 1, 3, and 7) demonstrate C_q_ values above 33.39. These samples are considered as false negatives. The negative result can either be caused by the ineffective enrichment of male CTs by the VTX‐1 Liquid Biopsy System, or by the stringent threshold C_q_ value of the qPCR assay. All C_q_ values are listed in Table 1. In Figure 2, the fluorescent signal of each sample is plotted against the cycle number.

**TABLE 1:** Overview of gestational age of the pregnant women at time of sample collection, confirmed fetal sex, C_q_ value after VTX‐1 enrichment, qPCR outcome, and estimated number of CTs, for all collected first trimester maternal blood samples

Sample	Gestational age	Fetal sex	C_q_	qPCR outcome	Estimated number of CTs
1	12w3d	Male	33.57	False negative	‐
2	12w6d	Male	31.67	True positive	6.2
3	11w3d	Male	33.95	False negative	‐
4	12w6d	Male	31.82	True positive	5.6
5	12w4d	Male	32.33	True positive	4.0
6	12w6d	Male	32.61	True positive	3.4
7	11w4d	Male	33.65	False negative	‐
8	12w2d	Male	32.35	True positive	4.0
9	13w3d	Male	32.43	True positive	3.8
10	11w5d	Male	32.87	True positive	2.9
11	12w2d	Female	33.24	True negative	‐
12	13w0d	Female	34.20	True negative	‐
13	12w4d	Female	34.49	True negative	‐
14	12w6d	Female	33.69	True negative	‐
15	13w3d	Female	33.54	True negative	‐

Four maternal blood samples were processed in duplicate on the VTX‐1 Liquid Biopsy System to allow both Y‐chromosome‐specific qPCR and Y‐chromosome STR analysis after enrichment. The presence of Y‐STR alleles confirms the presence of male DNA, as nonspecific amplification is rare for Y‐STR analysis. Two male alleles are detected in the STR profiles of samples 8 and 10, which confirms their true positive result, determined by qPCR. As expected, the true negative samples 14 and 15 showed empty Y‐STR profiles. Y‐STR profiles of samples 8 and 10 are shown in Supplementary Figure 2.

To evaluate the number of male CTs recovered by VTX‐1 enrichment, the amount of recovered male DNA after VTX‐1 processing is estimated using a standard curve measured on a dilution series of male reference DNA in a background of 8 ng female DNA as described in the methods section. Next, the corresponding number of enriched male trophoblasts is calculated, based on the assumption that each human diploid cell contains 6 pg of genomic DNA. All estimated numbers of enriched male CTs are listed for the true positive samples in Table 1. This estimation demonstrates a recovery between 2 and 6 CTs from 8 ml of maternal blood in seven out of ten first trimester samples.

### 3.2 Performance of VTX‐1 Liquid Biopsy System

The target cell recovery of VTX‐1 enrichment is estimated by means of spiking experiments with trophoblast cells from a JEG‐3 cell line. The results indicate that after VTX‐1 processing of whole blood spiked with pre‐stained JEG‐3 cells, on average 35.27 % ± 5.49 % (*n* = 6) of the target cells are retained. Individual results are listed in Supplementary Table 3. Since JEG‐3 cells are comparable, but not identical as trophoblasts, this result is an estimation for the trophoblast recovery.

The number of retained maternal cells is assessed based on the total amount of DNA, present after VTX‐1 enrichment. An average DNA yield of 9.15 ng ± 3.41 ng (*n* = 3) is obtained from 8 ml of blood, which corresponds with the presence of 1524 ± 569 cells.

## 4 DISCUSSION

Today, the most successful cbNIPT workflows are based on a combination of intra‐ or extracellular CT markers. However, this marker‐based approach necessitates several pre‐enrichment procedures, such as cell fixation and red blood cell lysis, inevitably resulting in a loss of target cells. To compensate for this loss, high volumes of blood are required, which may cause discomfort for the pregnant woman during collection. Moreover, these additional processing steps, result in a longer processing time and higher reagent costs. Therefore, in this paper, the VTX‐1 Liquid Biopsy System is assessed for the enrichment of CTs from maternal blood, based on laminar microscale vortices. This size‐based technology requires no pre‐enrichment steps, as whole blood can be directly processed on the Vortex chip.

Based on the results of the Y‐chromosome specific qPCR assay, in seven out of ten samples from women carrying a male fetus, male CTs were successfully enriched using the VTX‐1 Liquid Biopsy system, starting from 8 ml of maternal blood. To decide whether or not the qPCR signal was caused by the presence of male cells, a threshold C_q_ value was determined using samples of women carrying a female fetus. Unfortunately, relatively low C_q_ values were noted for these true negative samples, which could be attributed to non‐specific amplification of the high amount of background female DNA, present in each sample after VTX‐1 enrichment. Hence, the proposed threshold C_q_ value of 33.39 was relatively low, resulting in a small range between true positive and true negative results. Based on this determined threshold C_q_ value, the presence of male CTs after enrichment could not be proven in three out of ten samples, which were therefore classified as false negatives.

To confirm the qPCR results, Y‐STR analysis was performed on four random samples that were processed on the VTX‐1 Liquid Biopsy System in duplicate. This STR assay is originally intended to generate male DNA profiles from mixtures of male and female DNA up to a ratio of 1:4000 and is therefore well‐suited to detect male DNA in the pool of female cells after VTX‐1 enrichment. However, kit specifications mention that incomplete profiles can be obtained when the DNA input is below 100 pg. Hence, as VTX‐1 enrichment of 8 ml of blood results in male DNA yields around 30 pg, incomplete STR‐profiles are expected. Nevertheless, the four qPCR results were confirmed by Y‐STR analysis, as incomplete Y‐STR profiles were obtained for true positive samples 8 and 10, while true negative samples 14 and 15 resulted in empty Y‐STR profiles. The results of both assays are in concordance, which demonstrates the reliability of the qPCR assay. The qPCR assay was chosen as the preferred method to process all samples because it allows an estimation of the number of recovered CTs, despite the occurrence of non‐specific amplification in female samples.

A CT recovery below the qPCR threshold, which is reported for three samples in this study, can be attributed to two causes. First, the average target cell recovery of the VTX‐1 Liquid Biopsy System is determined at 35.27 % ± 5.49 %, which means that about 65 % of CTs are lost. Therefore, as the number of CTs varies among pregnant women, in some cases VTX‐1 enrichment might be unsuccessful. Second, only 8 ml of maternal blood is processed, while other reported state‐of‐the‐art CT isolation protocols start from 30 to 40 ml.^20^ ^,^ ^21^ Increasing the volume of blood will probably decrease the number of samples for which no sufficient numbers of CTs are enriched.

Finally, the number of retained maternal cells after VTX‐1 enrichment is estimated at ±1524 cells. This low number of contaminating maternal blood cells will definitely simplify the downstream scanning and selection procedure for final single trophoblast isolation in cbNIPT.

In a beginning‐to‐end cbNIPT workflow, VTX‐1 enrichment can be followed by fixation and intra‐ or extracellular staining in a collection recipient of choice. If needed, the stained cells can then be transferred to a microscopic glass slide and visualized using fluorescence microscopy. Finally, the target cells can be isolated individually, followed by whole genome amplification (WGA) and genetic analysis. Preliminary results of our research group indicate that VTX‐1 enriched cells maintain good quality and allow downstream staining with CD45/Cytokeratin/Hoechst. Moreover, DNA profiles can be obtained after single cell isolation using laser capture microdissection and WGA.

## 5 CONCLUSION

This proof‐of‐concept study shows that CTs can be enriched from maternal blood based on their larger cell size, using laminar microscale vortices. Y‐chromosome‐specific qPCR results indicate that CTs are recovered from 8 ml of maternal blood, in seven out of ten first trimester samples, using VTX‐1 enrichment. For these samples, the number of enriched CTs is estimated at 2–6 cells. For the other three samples, the presence of CTs could not be confirmed by qPCR. Only ±1524 maternal blood cells are retained after VTX‐1 enrichment, which simplifies subsequent CT isolation and cbNIPT analyses.

## CONFLICT OF INTEREST

The authors declare that they have no competing interests.

## Supporting information

Supplementary Material

## Data Availability

All data that supports the findings in this study is publicly available in the manuscript or the supplementary information.
