# Supplementary material for: Enrichment of circulating trophoblasts from maternal blood using laminar microscale vortices
Source: Prenat Diagn. 2021 Feb 1;41(9):1171–8. doi: 10.1002/pd.5901 (PMC8451878; doi:10.1002/pd.5901)

Enrichment of circulating trophoblasts from maternal blood using laminar microscale vortices

Running title: Size-based enrichment of circulating trophoblasts

Authors:

Ann-Sophie Vander Plaetsen[1,#], Jana Weymaere[1,#], Olivier Tytgat[1,2], Magaly Buyle[3], Dieter Deforce[1], Filip Van Nieuwerburgh[1,*]

Affiliations:

[1]Laboratory of Pharmaceutical Biotechnology, Ghent University, 9000 Gent, Belgium
[2]Department of Life Science Technologies, Imec, 3001 Leuven, Belgium.
[3]Obstetrics and gynecology, Ghent University Hospital, 9000 Gent, Belgium
[#] These authors contributed equally.
[*]Corresponding author (email: Filip.VanNieuwerburgh@UGent.be).

Corresponding author:

Prof. Filip Van Nieuwerburgh
Ghent University, Laboratory of Pharmaceutical Biotechnology
Ottergemsesteenweg 460, 9000 Gent, Belgium
email: Filip.VanNieuwerburgh@UGent.be

Supplementary Table 1. Individual and average C_q values used for standard curve

Sample	Individual C_q values			Average C_q ± SD
20 pg/µL male DNA + 8 ng female DNA	26.67	26.72	26.74	26.71 ± 0.03
10 pg/µL male DNA + 8 ng female DNA	27.77	27.81	27.66	27.75 ± 0.08
5 pg/µL male DNA + 8 ng female DNA	28.58	28.82	28.67	28.69 ± 0.12
2.5 pg/µL male DNA + 8 ng female DNA	29.66	29.55	29.40	29.54 ± 0.13
1.25 pg/µL male DNA + 8 ng female DNA	30.84	30.59	30.63	30.69 ± 0.13
0.63 pg/µL male DNA + 8 ng female DNA	31.22	31.74	32.03	31.66 ± 0.41
0.32 pg/µL male DNA + 8 ng female DNA	32.73	33.36	33.29	33.13 ± 0.35
0.16 pg/µL male DNA + 8 ng female DNA	33.93	34.40	34.82	34.38 ± 0.45
8 ng female DNA	35.33	48.84	41.80	41.99 ± 6.79
Water (NTC)	> 50	> 50	> 50	-

Supplementary Table 2. Plasma C_q values of all maternal blood samples.

Sample	C_q value	Fetal sex confirmed by Hospital
1		male
2	29.22	male
3	30.88	male
4	28.49	male
5	27.82	male
6	28.63	male
7	30.80	male
8	28.88	male
9	28.80	male
10	28.03	male
11	>35	female
12	>35	female
13	>35	female
14	>35	female
15	>35	female

Supplementary Table 3. Exact values of spiking experiments with JEG-3 cells for recovery calculation of VTX-1 enrichment.

# spiked cells	# recovered cells	Recovery (%)	Average (%) ± SD
118	40	33.90	35.27 ± 5.49
100	36	36.00	
21	7	33.33	
1145	482	42.10	
487	129	26.49	
3389	1349	39.81	

Supplementary Figure 1. Average standard curve, R^2, intercept, and slope for the Y-chromosome-specific qPCR assay. Average C_q values are plotted in function of male reference DNA concentration in a background of 8 ng female DNA.

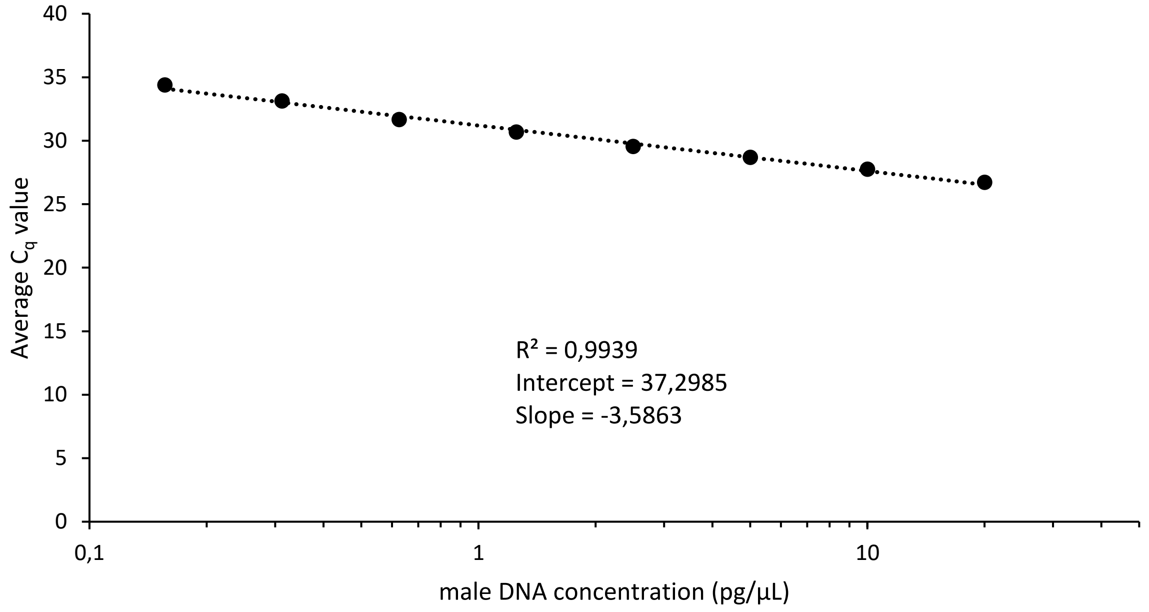

Supplementary Figure 2. Y-STR profiles of sample 8 and 10. Electropherograms show the relative fluorescence unit in function of the length in base pairs.

Sample 8

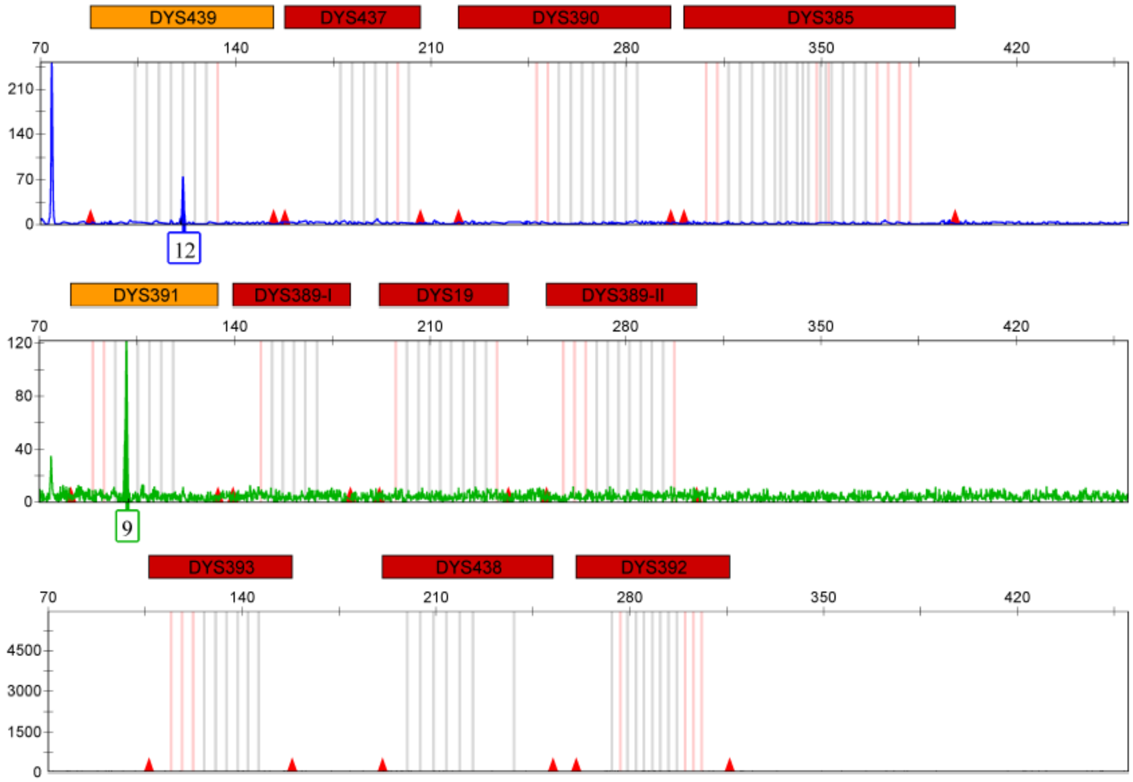

Sample 10

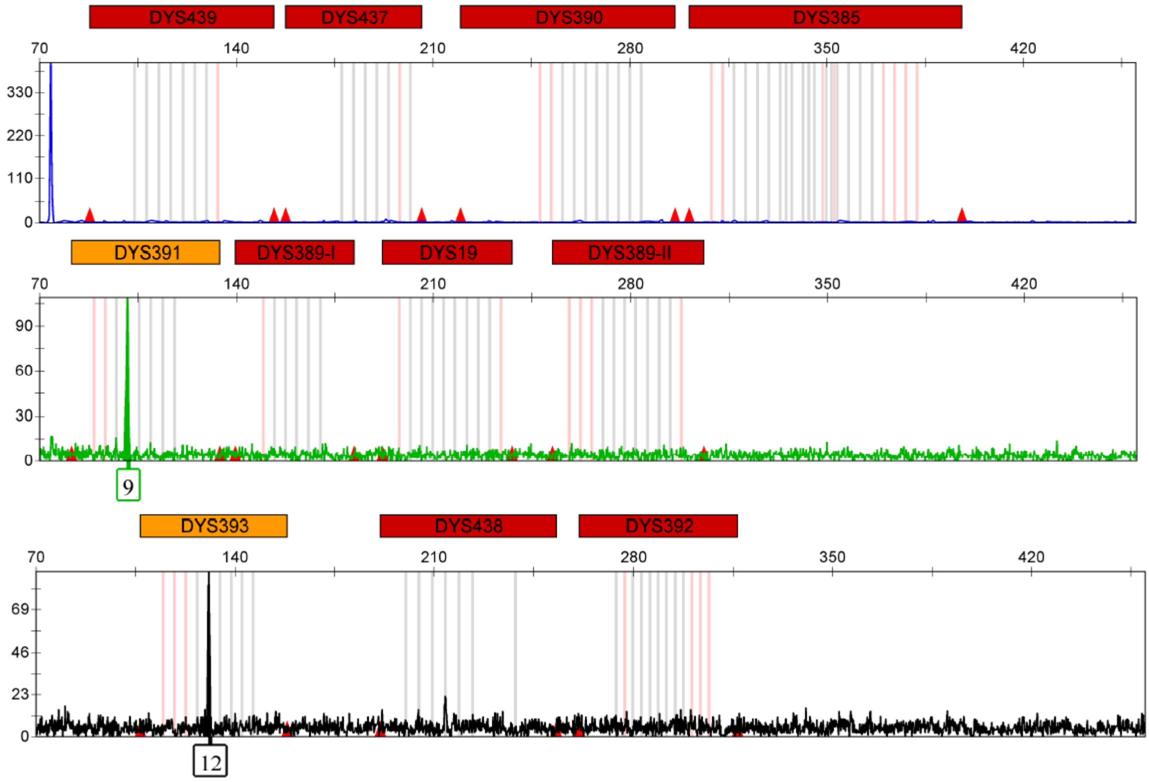

Supplement: Supplementary file 1 — Supplementary Material [file PD-41-1171-s001.pdf]
